# Evaluation of Telomerase Reverse Transcriptase (TERT) Promoter Mutations in Photodynamic Diagnosis With 5-Aminolevulinic Acid False-Positive Sites in Non-muscle-Invasive Bladder Cancer

**DOI:** 10.7759/cureus.90860

**Published:** 2025-08-24

**Authors:** Yoshitaka Saito, Shogo Adomi, Kazuko Sakai, Marco Antonio De Velasco, Mamoru Hashimoto, Eri Banno, Yujiro Hayashi, Takafumi Minami, Kazuto Nishio, Kazutoshi Fujita

**Affiliations:** 1 Urology, Kindai University Hospital, Osakasayama, JPN; 2 Urology, Mimihara General Hospital, Sakai, JPN; 3 Genome Biology, Kindai University Hospital, Osakasayama, JPN; 4 Urology, Johns Hopkins University, Baltimore, USA

**Keywords:** 5-aminolevulinic acid, bladder cancer, photodynamic diagnosis, tert promoter mutation, turbt

## Abstract

Background/aim: Photodynamic diagnosis (PDD) with 5-aminolevulinic acid (5-ALA) can be used to detect bladder cancer lesions, but false-positive results without pathological malignancy could sometimes occur. However, a false-positive lesion might be the precancerous lesion. Telomerase reverse transcriptase (*TERT*) promoter mutations are commonly found in the tumors and urine of bladder cancer patients. *TERT *promoter mutations have been detected in normal mucosa and have been associated with subsequent bladder cancer development. In this study, we evaluated *TERT* promoter mutations in PDD false-positive samples in patients with non-muscle-invasive bladder cancer (NMIBC) who underwent PDD-assisted transurethral resection of bladder tumor (PDD-TURBT) after oral 5-ALA administration.

Materials and methods: Among 117 patients who underwent PDD-TURBT for NMIBC, 18 NMIBC patients who had PDD with 5-ALA positive samples, which were pathologically diagnosed as no malignant diseases, were included in this study. *TERT* promoter mutations were evaluated in 24 PDD false-positive samples. Droplet digital PCR (ddPCR) was employed to detect C228T and C250T mutations in the* TERT* promoter region using genomic DNA. The relationship between mutations in the* TERT* promoter and recurrence of bladder tumors was assessed using the Kaplan-Meier survival analysis.

Results: *TERT* promoter mutations in PDD false positives were found in six (33.3%) patients. C228T mutation was found in five (27.8%) patients, and C250T mutation was found in one (5.5%) patient. *TERT* promoter mutations were significantly associated with the presence of two or more tumors (P=0.033) and with an European Organisation for Research and Treatment of Cancer (EORTC) risk score of 5 or higher (P=0.010) in PDD false-positive cases. Two-year recurrence-free survival (RFS) rate was 33.3% in *TERT *promoter mutation-positive cases and 50% in negative cases. There was no significant difference in RFS between *TERT* promoter-positive cases and negative cases (P=0.698).

Conclusions: *TERT* promoter mutations were frequently found in PDD false-positive samples in patients with NMIBC who underwent PDD-TURBT. While limited in extent, PDD false-positive samples in patients with *TERT* promoter mutation positivity in the non-malignant urothelium might be precancerous lesions.

## Introduction

Non-muscle-invasive bladder cancer (NMIBC) accounts for 75% of bladder cancers [1]. NMIBC recurs in 50%-80% of cases, with disease progression in 10%-15% [2,3]. The standard treatment for NMIBC is transurethral resection of bladder tumor (TURBT). pT1, high-grade bladder cancer, and high-risk NMIBC concurrent with carcinoma in situ (CIS) are associated with residual tumor at TURBT [4,5]. Recently, photodynamic diagnosis (PDD) with 5-aminolevulinic acid (5-ALA) has been used at TURBT to decrease the insufficient dissection of the tumor [4]. PDD-TURBT takes advantage of the way protoporphyrin IX, a naturally fluorescent compound, tends to gather in cancerous cells [6]. In NMIBC, fluorescent cystoscopy combined with PDD allows the observation of cancer lesions by fluorescent emission, making it a useful diagnostic method [7]. Furthermore, PDD-TURBT is a more efficient method than conventional white light TURBT (WL-TURBT) and has been shown to increase tumor detection rates and decrease the risk of disease recurrence [8,9]. Mutations in the promoter region of the telomerase reverse transcriptase (*TERT*) gene are commonly found in many primary tumors, such as bladder cancer and its early-stage lesions [10-12]. Furthermore, mutations in the upstream promoter of *TERT* C228T and C250T were mainly affected [13]. *TERT* promoter mutations result in increased expression of *TERT*, permitting malignant cells to maintain telomere shortening without triggering genetic senescence [14]. In addition, *TERT* promoter mutations cause tumorigenesis by promoting genomic instability [15]. Mutations in the *TERT* promoter region, particularly at the C228T and C250T hotspots, have been linked to higher rates of recurrence and progression in NMIBC [16,17]. Using liquid biopsy technology, the detection of *TERT *promoter mutations in urinary cell-free and cellular DNA is now feasible even before cystoscopy reveals any signs of urothelial carcinoma [18,19]. Furthermore, *TERT* promoter mutations were found in 9% of bladder biopsies with the normal bladder mucosa [20]. Therefore, *TERT* promoter mutations in the bladder mucosa could be the initiation of bladder cancer development. PDD-TURBT could sometimes result in a diagnosis of no histological malignancy (false positive) with the positive fluorescent emitted sites. False-positive findings in PDD were defined as lesions exhibiting marked red fluorescence under blue light during rigid cystoscopy after oral 5-ALA administration, with no histopathological evidence of bladder cancer. These false-positive sites might be a precancerous lesion.

This study is the first to demonstrate an association between *TERT* promoter mutations and PDD false-positive sites, and it further aims to assess the potential of *TERT* promoter mutations as predictors of bladder cancer recurrence. Therefore, we analyzed *TERT* promoter mutations in PDD false-positive sites in NMIBC patients who underwent PDD-TURBT.

## Materials and methods

**Figure 1:**
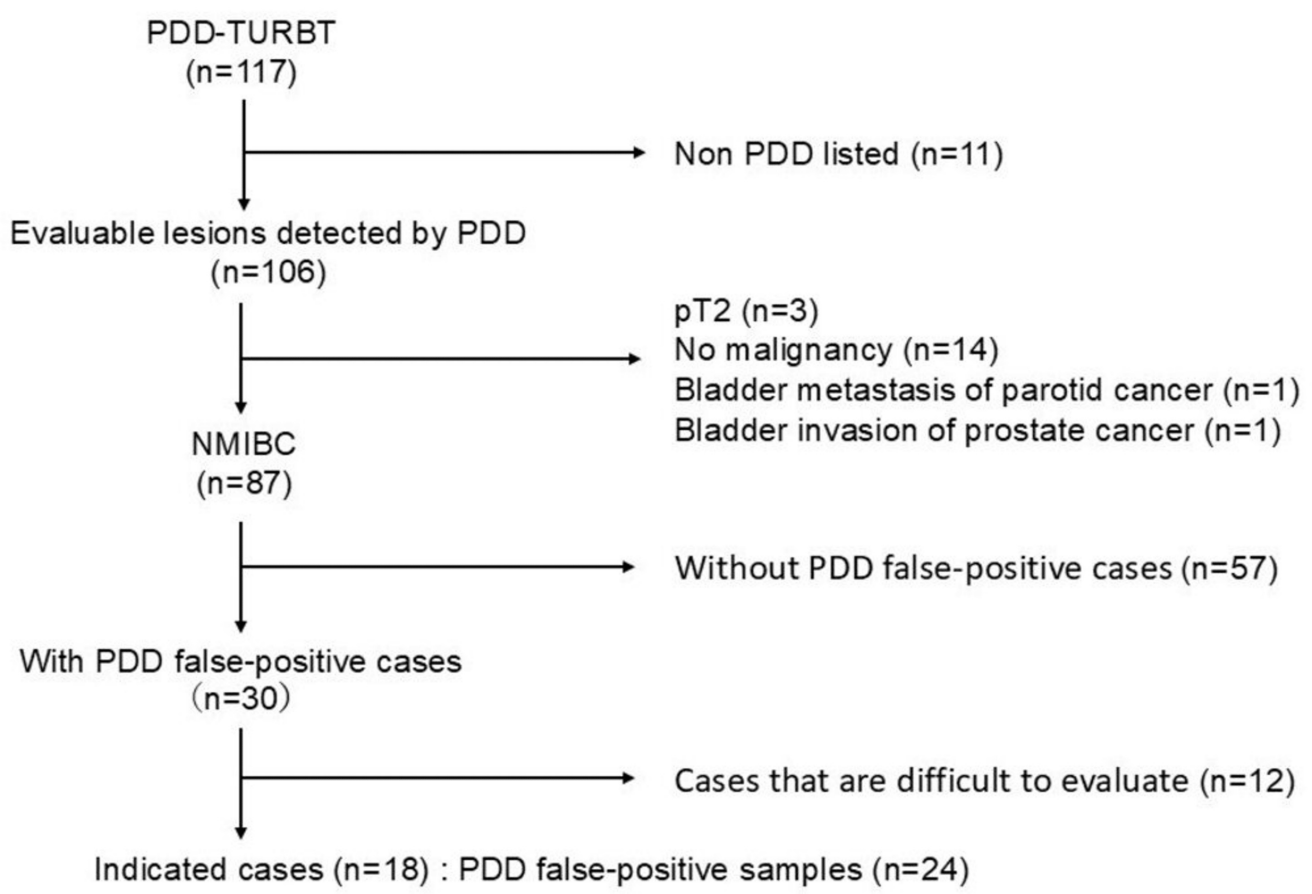
Patient selection Among 117 patients who underwent PDD-TURB, 18 NMIBC patients and 24 samples who had PDD with positive samples, which were pathologically diagnosed as no malignant diseases, were included. PDD-TURBT: photodynamic diagnosis-transurethral resection of bladder tumor, NMIBC: non-muscle-invasive bladder cancer

This study included 117 patients who underwent PDD-TURBT after oral 5-ALA administration at Kindai University Hospital from July 2011 to August 2023. Of the 117 patients, those with pT2 or higher bladder metastases from non-urothelial cancer, bladder invasion from prostate cancer, no false-positive PDD, no bladder cancer, and sections that were difficult to evaluate were excluded. Eighteen patients with PDD false-positive results were included in the study (Figure 1).

Tumor samples were obtained from formalin-fixed paraffin-embedded (FFPE) samples. A total of 42 samples were taken from 18 patients who underwent PDD-TURBT. The number of slide sections required per sample was 10-15 sections at 5 micron thickness. In addition, 3-5 sections were used for a 10 mm × 10 mm area and 10 or more for a 2 mm × 2 mm area, depending on the size of the tissue.

Pathological diagnosis

At least two experienced pathologists were involved in making the histological assessments. Tumor staging and grading followed the guidelines outlined in the eighth edition of the American Joint Committee on Cancer (AJCC) Cancer Staging Manual [21]. Tumor grading was performed based on the 2016 guidelines established by the World Health Organization (WHO) [22].

DNA extraction

DNA was extracted and purified from the formalin-fixed paraffin-embedded (FFPE) tissue samples using the GeneRead DNA FFPE Kit (QIAGEN, Hilden, Germany), following protocols described in earlier studies [23]. The quality and quantity of the DNA were verified using a NanoDrop 2000 device (Thermo Fisher Scientific, Waltham, MA) and PicoGreen dsDNA assay kit (Thermo Fisher Scientific). The extracted DNA were stored at -80°C until further analysis.

Droplet digital PCR assay (ddPCR)

To detect *TERT* promoter mutations, we used the ddPCR platform QX100 Droplet Digital PCR System (Bio-Rad Laboratories, Hercules, CA), including primers and probes (FAM, mutant type; HEX, wild type), and ddPCR Supermix for Probes (no dUTP), according to the manufacturer’s protocol. The specific primers and probes used for ddPCR are as follows: assay ID: dHsaEXD72405942 (C228T) and dHsaEXD72405950 (C250T). The droplets were generated using a droplet generator (Bio-Rad Laboratories). We used 60 ng (range: 4.2-60 ng) of FFPE DNA and added 5 mM betaine and 1 mM ethylenediaminetetraacetic acid (EDTA) to each sample, followed by PCR. The PCR protocol consisted of an initial denaturation at 95°C for 10 minutes, 40 cycles of 94°C for 30 seconds and 60°C for one minute, enzyme inactivation at 98°C for 10 minutes, and holding at 10°C. After the PCR reaction, the samples were transferred to a droplet reader (Bio-Rad Laboratories). Digital PCR data were analyzed using the Quanta Soft analytical software package (Bio-Rad). The samples were considered positive for targeted mutations when they met two criteria:(i) they contained at least three droplets in the positive area for the FAM signal and (ii) the minor allele frequency (MAF) was more than 0.5%. MAF was defined as the ratio of mutant-type copies to the total number of mutant and wild-type copies obtained by the ddPCR platform, as previously reported [23].

Sample size

As an initial validation step, a preliminary study was conducted using 24 samples, taking into account resource limitations (time and budget) and ethical considerations. The sample size was set as the minimum required to evaluate both the detectability of the biomarker and technical reproducibility. Specifically, assuming a mutation frequency of 30% and a ddPCR technical error with a standard deviation of 10%, a priori power analysis using G*Power confirmed that 20-25 samples would be sufficient to detect a statistically significant difference at α = 0.05 with 80% power. Regarding the technical protocol, DNA was independently extracted and quantified using Qubit for each sample, followed by ddPCR analysis with the Bio-Rad QX100 system. On average, more than 15,000 droplets were generated per reaction, allowing for highly reliable quantification. A sample was considered positive when three or more droplets exceeded the predefined fluorescence threshold. In this study, we confirmed the technical feasibility of mutation detection using ddPCR with 24 samples as part of the initial validation phase. For future studies, we plan to expand the sample size to further enhance statistical power. Moreover, the tissue area, DNA quantity, and reaction conditions were all optimized to balance reproducibility and sensitivity, ensuring a detection limit of at least 0.5% mutant allele frequency. Based on the above, the experimental conditions have been optimized to achieve a balance between reproducibility and sensitivity.

Statistical analysis

Statistical analysis was performed using StatMate V software (ATMS Co., Ltd., Tokyo, Japan). Chi-square and Fisher’s exact tests were performed to evaluate the clinical, pathological, and PDD false-positive sites of *TERT* promoter. The association between *TERT *promoter mutations and bladder cancer recurrence was examined using the Kaplan-Meier method, and statistical analysis was conducted using the log-rank test. A p-value of <0.05 was considered to be statistically significant.

## Results

*TERT* promoter mutations in tumors

Samples from 18 patients who underwent PDD-TURBT but were pathologically diagnosed as no malignant (PDD false positive) were included in this study. All 18 patients had PDD false-positive samples, which were positive for PDD-TURBT but pathologically diagnosed as no malignant. Table 1 summarizes the patients’ characteristics.

**Table 1:** Patients’ characteristics stratified with TERT promoter mutations status in NMIBC cases *p-values for categorical variables are derived from Chi-square and Fisher’s exact tests. p<0.05 indicates statistical significance. NMIBC: non-muscle-invasive bladder cancer, CIS: carcinoma in situ, EORTC: European Organisation for Research and Treatment of Cancer, *TERT*: telomerase reverse transcriptase

Factors	All	*TERT* promoter mutations (positive)	*TERT* promoter mutations (negative)	*p-value	Degrees of freedom	Effective size
	Number	Number (%)	Number (%)			
	18	9 (50)	9 (50)			
Age						
Median (range)	78	80 (70-86)	74 (34-84)	0.27	8	0.086
Sex						
Male	13	7 (77.8)	6 (66.7)	0.598	1	0.124
Female	5	2 (22.2)	3 (33.3)			
Smoking history						
Yes	13	6 (66.7)	7 (77.8)	0.598	1	0.124
No	5	3 (33.3)	2 (22.2)			
Number of tumors						
Single	12	5 (55.6)	7 (77.8)	0.247	2	0.393
2-7	5	4 (44.4)	1 (11.1)			
≥8	1	0 (0)	1 (11.1)			
Tumor diameter						
<30 mm	16	8 (88.9)	8 (88.9)	0.453	1	0.176
≥30 mm	2	1 (11.1)	1 (11.1)			
Prior recurrence						
Primary	7	2 (22.2)	5 (55.6)	0.059	2	0.56
≤1 year recurrence	6	6 (66.7)	1 (11.1)			
>1 year recurrence	5	1 (11.1)	3 (33.3)			
Pathological T stage						
pTa	13	8 (88.9)	5 (55.6)	0.124	2	0.326
pT1	5	1 (11.1)	4 (44.4)			
Concurrent CIS	4	2 (22.2)	2 (22.2)			
Grade						
G1	8	3 (33.3)	5 (55.6)	0.661	2	0.212
G2	5	3 (33.3)	2 (22.2)			
G3	5	3 (33.3)	2 (22.2)			
EORTC						
Low	3	1 (11.1)	2 (22.2)	0.138	2	0.661
Intermediate	14	8 (88.9)	6 (66.7)			
High	1	0 (0)	1 (11.1)			

**Figure 2:**
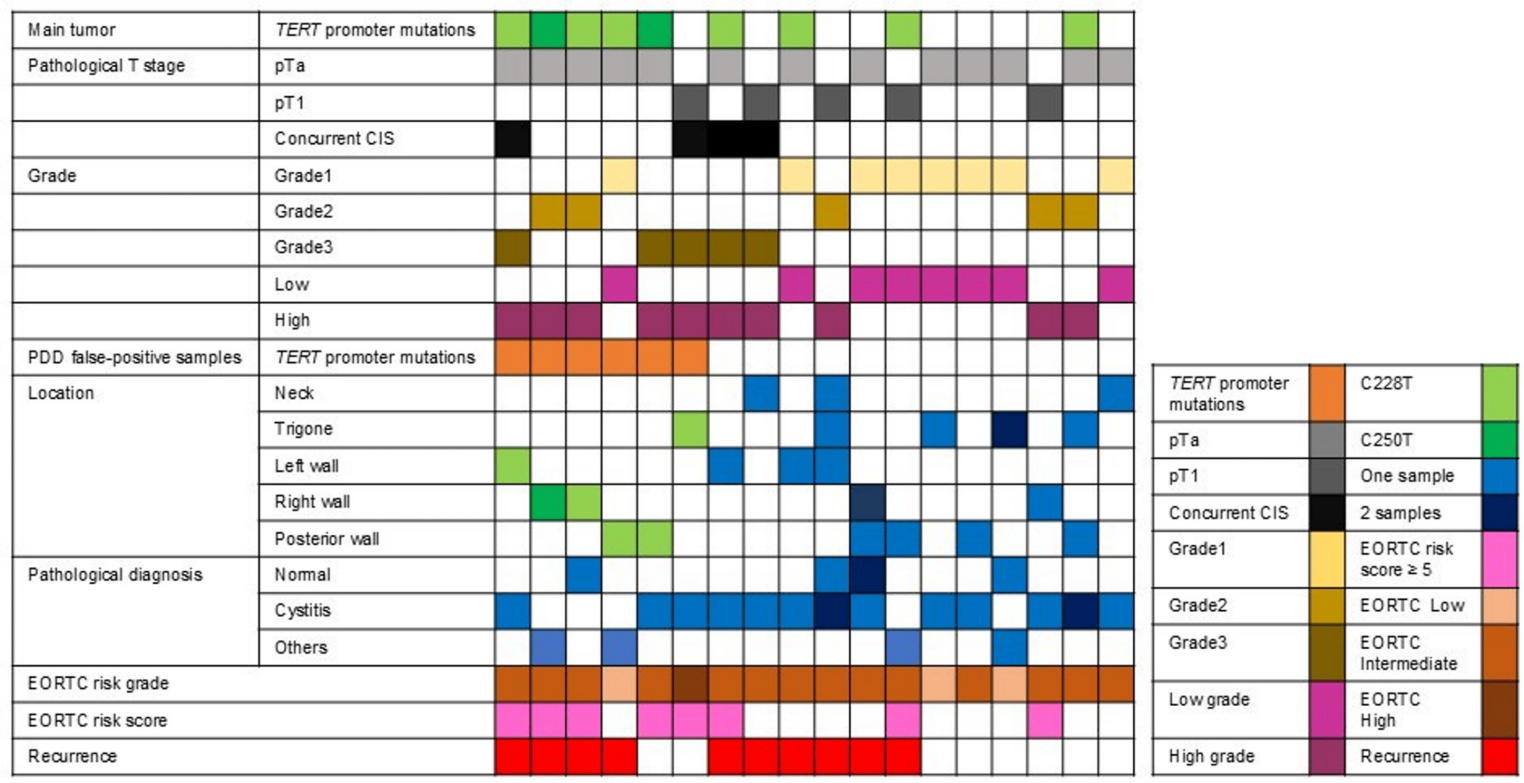
Association of main tumor status and TERT promoter mutations in PDD false-positive cases Association of pathological, location, and clinical diagnosis of TERT promoter mutations in the main tumor and PDD false-positive samples. Others were urothelial papilloma, epithelioid granuloma, Brunn’s nest, and PUNLMP. CIS: carcinoma in situ, 5-ALA: 5-aminolevulinic acid, EORTC: European Organisation for Research and Treatment of Cancer, PUNLMP: papillary urothelial neoplasm of low malignant potential, TERT: telomerase reverse transcriptase

The median age of the patients was 78 (range: 34-86) years. There is no significant difference between *TERT* promoter mutation-positive and TERT promoter mutation-negative cases in NMIBC patients. Among 18 NMIBC samples, nine (50%) were positive for *TERT* promoter mutations, in which seven (38.9%) samples had C228T mutation and two (11.1%) had C250T mutation, while nine (50%) NMIBC samples were negative for *TERT* promoter mutations (Figure 2).

*TERT* promoter mutations in PDD false-positive samples

From 18 patients, 24 PDD false-positive samples were analyzed. Among 18 patients, six (33.3%) had PDD false-positive samples with *TERT* promoter mutation (Figure 2). Six PDD false-positive samples from six (33.3%) patients were positive for* TERT* promoter mutations, in which five (27.8%) had C228T mutation and one (5.5%) had C250T mutation. The presence of *TERT* promoter mutations in PDD false-positive samples were not statistically associated with age, gender, smoking history, tumor size, time to recurrence, pathological T classification, concurrent CIS, and grade. In contrast, two or more tumors and an EORTC risk score of 5 or higher were significantly associated with the presence of *TERT* promoter mutations in PDD false-positive cases (Chi-square and Fisher’s exact tests, P=0.033, P=0.010) (Table 2).

**Table 2:** Risk factors of TERT promoter mutations positive in PDD false-positive cases *p-values for categorical variables are derived from Chi-square and Fisher’s exact tests. p<0.05 indicates statistical significance. NA: not available, 5-ALA: 5-aminolevulinic acid, CI: confidence interval, CIS: carcinoma in situ, EORTC: European Organisation for Research and Treatment of Cancer

Factors	Odds ratio (95% CI)	*p-value	Degrees of freedom	Effective size
Age				
≥79 versus ≤78	2.0 (0.260-15.38)	0.614	1	0.118
Sex				
Male versus female	0.666 (0.078-5.678)	0.576	1	0.131
Smoking				
Yes versus no	2.5 (0.213-29.25)	0.576	1	0.131
Tumor size				
≥30 mm versus ≤29 mm	NA	0.111	1	0.375
Tumor number				
Multiple versus single	10 (1.025-97.50)	0.033	1	0.500
Duration to recurrence (year)				
≤2 versus >2	2.0 (0.260-15.38)	0.614	1	0.118
Pathological T stage				
pT1 versus pTa	0.4 (0.034-4.680)	0.576	1	0.131
Concurrent CIS				
Positive versus negative	2.5 (0.256-24.37)	0.546	1	0.141
Grade				
G3 versus G1 and G2	5.0 (0.550-45.39)	0.264	1	0.263
High versus low	7.0 (0.613-79.87)	0.131	1	0.355
EORTC				
≥5 versus <4	25 (1.802-346.7)	0.010	1	0.604

Since the neck and trigone were known to be false-positive sites of PDD [24], we analyzed the association of *TERT* promoter mutations and the location of false-positive samples. One sample was located at the trigone and left wall, and two samples were at the right wall and the posterior wall (Figure 2). Comparing the samples from the neck or trigone with those from other sites, there is no significant difference in *TERT* promoter mutations in the sites other than the neck and trigone (Chi-square and Fisher’s exact tests, P=0.330) (Table 3).

**Table 3:** Association of location and pathological diagnosis with TERT promoter mutations in PDD false-positive samples Others were urothelial papilloma, epithelioid granuloma, Brunn’s nest, and PUNLMP. *p-values for categorical variables are derived from Chi-square and Fisher’s exact tests. p<0.05 indicates statistical significance. 5-ALA: 5-aminolevulinic acid, CI: confidence interval, PUNLMP: papillary urothelial neoplasm of low malignant potential, *TERT*: telomerase reverse transcriptase

Factors	*TERT* promoter mutations (positive)	*TERT* promoter mutations (negative)	*p-value	Degrees of freedom	Effective size
	Number (%)	Number (%)			
	6 (25)	18 (75)			
Location					
Neck and trigone	1 (16.7)	8 (44.4)	0.330	1	0.198
Other than the neck and trigon	5 (83.3)	10 (55.6)			
Pathological diagnosis					
Normal	1 (16.7)	4 (22.2)	0.542	2	0.225
Cystitis	3 (50)	12 (66.7)			
Others	2 (33.3)	2 (11.1)			

Among six PDD false-positive samples with the presence of *TERT* promoter mutations, normal urothelial mucosa was found in one (16.7%), cystitis in three (50%), and others (epithelioid granuloma and papillary urothelial neoplasm of low malignant potential) in two (33.3%). There was no association between the pathology of PDD false-positive samples and the presence of *TERT* promoter mutations (Chi-square and Fisher’s exact tests, P=0.542) (Table 3).

Association of *TERT* promoter mutations in the PDD false-positive site with intravesical tumor recurrence

**Figure 3:**
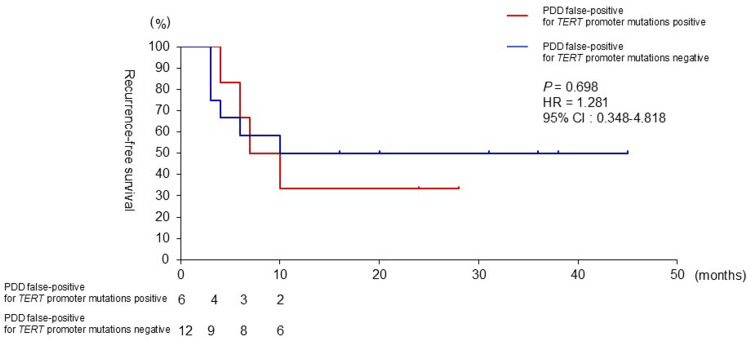
Recurrence-free survival in bladder cancer cases PDD false-positive for TERT promoter mutations (positive and negative) Kaplan-Meier curves for recurrence-free survival in bladder cancer patients PDD false-positive for *TERT* promoter mutations (positive and negative). Statistical analysis was performed using the log-rank test. PDD: photodynamic diagnosis, *TERT*: telomerase reverse transcriptase

The median follow-up period for 18 patients was 10 months (3-45). Ten (55.6%) patients experienced intravesical recurrence. Four of six (66.6%) patients with *TERT* promoter mutations in PDD false-positive samples experienced recurrence, while six of 12 (50%) patients without *TERT* promoter mutations in PDD false-positive samples experienced recurrence (Chi-square and Fisher’s exact tests, P=0.614). The two-year recurrence-free survival was 33.3% in patients with positive *TERT* promoter mutations and 50% in patients with negative TERT promoter mutations. The status of *TERT* promoter mutations in PDD false-positive patients were not associated with intravesical recurrence (log-rank test, P=0.698) (Figure 3).

## Discussion

In this study, TERT promoter mutations in PDD false-positive sites were found in 33.3% of NMIBC patients who underwent PDD-TURBT. The recurrence rates between those positive for TERT promoter mutations and those negative were 66.6% and 50%, respectively, although there was no significant difference. False positives in PDD-TURBT have been reported to be 17.4% [24]. False positives can result from tangent effects or inflammation [24,25]. PDD false-positive tissue is not classified as cancerous tissue in pathological diagnosis, but some of these have been reported to contain precancerous lesions [26]. In addition, there are no reports demonstrating genetic alterations in PDD-TURBT false-positive sites.

In our study, we could not prove with a significant difference that PDD false positive samples in patients with *TERT* promoter mutation positivity in the non-malignant urothelium increased the intravesical recurrence and precancerous lesions. On the other hand, the *TERT *promoter mutation positivity rate for PDD false positivity was 33.3%. These results might suggest that the false positivity of PDD-TURBT with 5-ALA is partially due to *TERT* promoter mutations. Furthermore, the presence of *TERT* promoter mutations in the PDD false-positive sites of PDD-TURBT using 5-ALA was considered to be a possible precancerous lesion of bladder cancer. *TERT* promoter mutations lead to elevated telomerase expression, enabling various cancers to overcome the end-replication problem and evade senescence. Mutations in the *TERT* promoter are the most commonly observed genetic alterations across all stages and grades of bladder cancer [10-12]. We have reported that the presence of the *TERT* promoter mutation (C228T) in non-malignant bladder urothelium is significantly associated with a higher recurrence rate of bladder cancer [20]. Therefore, for PDD false-positive samples in patients with *TERT* promoter mutations, it may be possible to recommend intravesical Bacillus Calmette-Guérin (BCG) therapy for recurrence prevention, as well as immediately after TURBT intravesical instillation of pirarubicin in the event of early recurrence. Additionally, long-term cystoscopic surveillance for more than five years may be advisable even in pTa with low-grade bladder cancer. These findings suggest that patients with *TERT* promoter mutation positivity in the non-malignant urothelium may require long-term follow-up. Over 70% of Ta and T1 tumors carry these mutations, which are primarily concentrated at two hotspot sites located 124 and 146 base pairs upstream of the ATG start codon [27]. Detection of these mutations in 60% of CIS cases indicates that they likely represent a common and early occurrence in the pathogenesis of both NMIBC and MIBC [13]. Although mutations in the *TERT* promoter represent an early event in the onset of bladder cancer, its promoter hypermethylation appears to be a more dynamic process involved in the advancement of the disease. Somatic mutations in the *TERT* promoter region were found to be the most common, occurring in 44% of tumor samples.

Recurrence of bladder cancer has been linked to several genetic mutations. In this study, we only evaluated the presence of *TERT* promoter mutations. Regarding other genetic alterations, *FGFR3* mutations were detected in both low-grade and high-grade NMIBC, as well as in normal urothelial tissue. In contrast, *TP53* and* PIK3CA* mutations were exclusively found in tumor samples. *CDKN2A* mutations were observed in high-grade NMIBC and normal urothelium but were absent in low-grade NMIBC [18]. Since somatic mutations in the *TERT* promoter and *FGFR3* have been found in normal urothelium and are commonly reported in tumor tissues by The Cancer Genome Atlas (TCGA), they are considered potential driver mutations in the development of both low-grade and high-grade NMIBC. We believe that it will be necessary to include additional analyses of genetic alterations such as *FGFR3* and *TP53* in future studies.

We have reported that *TERT* C228T mutation was detected in 9% of normal bladder epithelium samples and in 27% of cases with urothelial dysplasia [20]. In this study, *TERT* promoter mutations at PDD false-positive sites were found in 33.3% of NMIBC patients, *TERT* C228T mutation in 27.8%, and *TERT* C250T mutation in 5.5%. We have previously shown that the presence of *TERT* promoter mutations in non-cancerous urothelium was significantly linked to the recurrence of bladder tumors within the bladder following TURBT [20]. Finally, PDD-TURBT might prevent intravesical recurrence by dissecting the precancerous lesion with PDD false-positive sites with *TERT* promoter mutations.

This study had a few limitations. First, it was conducted retrospectively and involved a limited number of cases. We could not perform multivariate analysis due to the small number of cases. Second, the median follow-up period was short (10 months), with a lack of statistically significant association with recurrence. We did not analyze gene mutations other than those in the *TERT* promoter, such as *FGFR3* and *TP53* [18,20]. These genes may affect the PDD with 5-ALA false positivity. Therefore, we would like to consider increasing the number of cases and extending the follow-up period, and as a future research direction, we plan to increase the number of cases through a multi-institutional collaborative study and to investigate not only *TERT* but also *FGFR3*, *TP53*, and other genetic mutations in PDD false-positive sites.

## Conclusions

*TERT* promoter mutation was frequently found in PDD false-positive samples in patients with NMIBC who underwent PDD-TURBT. While limited in extent, considering the rate of PDD false-positive samples in patients with *TERT* promoter mutation positivity, PDD false positives with 5-ALA in the non-malignant urothelium might be precancerous lesions.
